# Variable Selection of Spatial Logistic Autoregressive Model with Linear Constraints

**DOI:** 10.3390/e24111660

**Published:** 2022-11-15

**Authors:** Yunquan Song, Yuqi Su, Zhijian Wang

**Affiliations:** School of Science, China University of Petroleum, Qingdao 266580, China

**Keywords:** spatial logistic autoregressive model, linear constraint, variable selection, maximum likelihood

## Abstract

In recent years, spatial data widely exist in various fields such as finance, geology, environment, and natural science. These data collected by many scholars often have geographical characteristics. The spatial autoregressive model is a general method to describe the spatial correlations among observation units in spatial econometrics. The spatial logistic autoregressive model augments the conventional logistic regression model with an extra network structure when the spatial response variables are discrete, which enhances classification precision. In many application fields, prior knowledge can be formulated as constraints on the parameters to improve the effectiveness of variable selection and estimation. This paper proposes a variable selection method with linear constraints for the high-dimensional spatial logistic autoregressive model in order to integrate the prior information into the model selection. Monte Carlo experiments are provided to analyze the performance of our proposed method under finite samples. The results show that the method can effectively screen out insignificant variables and give the corresponding coefficient estimates of significant variables simultaneously. As an empirical illustration, we apply our method to land area data.

## 1. Introduction

Spatial econometrics, developed to deal with spatial correlation and spatial heterogeneity of data, has become a standard analytical tool for spatial data and has begun to enter the mainstream of econometrics. Spatial models have a long history in econometrics. Much progress has been made in the estimation of spatial models, please refer to the special works of Anselin (1988) [1]; LeSage and Pace (2009) [2]. Nowadays, the spatial autoregressive (SAR) model developed by Cliff and Ord in 1973 [3] is the most studied and widely applied modeling method for dealing with spatial correlation. Additionally, the model can be widely applied in many fields including social networks (Ma et al. (2020) [4]), real estate (Dubin (1999) [5]; Osland (2010) [6]), crime incidents (Kakamu et al. (2008) [7]), sampled network data (Zhou et al. (2017) [8]), artificial neural networks (Wang et al. (2019) [9]), and geospatial data (Khalfi et al. (2021)). We can consider the spatial autoregressive model as an ordinary regression model that additively takes the spatial spillover effect of the dependent variable into account. Thus, this model can model both traditional covariates and network dependence simultaneously in a convenient manner.

However, most of the conventional spatial analyses were designed to address the problem of estimation or prediction based on continuous observations. In the case of discrete or binary variables, for instance, in pathological diagnosis, there are only two possible outcomes, positive (denoted as 1) or negative (denoted as 0). The logistic regressive model is a popular method to deal with discrete binary responses. As one of the most popular classification methods, the logistic regressive model has been studied extensively. Essentially, the model assumes that an individual’s class label is influenced by a set of predictors. In practical use, observational data can be taken from different places. In other words, the data generated can be cross-section data. The cross-section data involve several locations. Therefore, it is possible that spatial effect influences the model. In the presence of spatial effects, the usual logistic regression does not sufficiently model the data. Thus, the spatial logistic regression model will be better to model data that contains spatial effects [10]. Theoretical economists and practical researchers are interested in the spatial logistic autoregressive model, which investigates how covariates affect the correlation response of spatial discrete values. The study of spatial logistic regression models, which use categorization technologies to model spatial data, is a relatively new area of spatial econometrics, and research in this area is still quite restricted. Calabrese and Elkink (2014) [11] introduced the binary spatial autoregressive model for the first time. Hilwin Nisa et al. (2019) [12] proposed the spatial logistic regression model which is obtained by the logistic regression model and spatial binary regression model.

High-dimensional spatial data appear frequently in many fields of social life and scientific research, such as biomedical imaging, X-ray tomography, finance, and geoscience. In recent years, a variety of regression methods have been proposed to model high-dimensional data in spatial statistics. For example, Piribauer et al. (2016) [13] proposed a Bayesian variable selection procedure in a spatial autoregressive model. A penalized quasi-maximum likelihood method was put forth by Liu et al. (2018) [14] for variable selection in the spatial autoregressive model. Model selection in spatial autoregressive models with varying coefficients was studied by Wei et al. (2019) [15]. Variable selection for the spatial autoregressive models with a diverging number of parameters was considered by Xie et al. (2020) [16]. Cai et al. (2020) [17] considered variable selection and estimation for a high-dimensional spatial autoregressive model. Li et al. (2020) [18] proposed a variable selection method for the partially linear varying coefficient spatial autoregressive model. More recently, Li et al. (2021) [19] proposed a new variable selection method for a higher-order partially linear spatial autoregressive model with a diverging number of parameters. Liu et al. (2021) [20] studied variable selection for the spatial autoregressive model with autoregressive disturbances. Song et al. (2021) [21] proposed a new robust variable selection method with an exponential squared loss for the spatial autoregressive model.

The above methods mainly focus on the variable selection of continuous response variables based on the penalty regression technique. Penalized regression techniques shrink the insignificant coefficients to 0, which has attracted increasing attention to high-dimensional data analysis, such as least absolute shrinkage and selection operator (LASSO) (Tibshirani (1996) [22]), smoothly clipped absolute deviation (SCAD) (Fan and Li (2001) [23]), and minimax concave penalty (MCP) (Zhang (2010) [24]) for mean regression. LASSO minimizes the residual sum of squares subject to the sum of the absolute value of the coefficients being less than a constant, for which it tends to produce some coefficients that are exactly 0 and hence give interpretable models. However, LASSO has some bias in the estimation of the coefficients. SCAD attempts to mitigate this bias and produce nearly unbiased estimates for large coefficients while still retaining the continuous penalty for sparsity. MCP provides the convexity of the penalized loss in sparse regions to the greatest extent given certain thresholds for variable selection and unbiasedness. However, penalized spatial logistic autoregression has been rarely studied. For high-dimensional spatial data, there are several problems using spatial logistic autoregressive modeling such as endogeneity and including too many variables in the model. First, the spatial lag term in the model will make it endogenous. In the presence of endogeneity, the ordinary least squares (OLS) method can produce biased and inconsistent parameter estimates. Second, as the dimensionality of the variables increases, redundant variables will bring challenges to the estimation in the modeling process.

Furthermore, as the penalized spatial logistic regression does not account for any prior information, and then we can consider how to incorporate prior information into the modeling procedure. Statistical models with linear constraints on variables have gained widespread applications recently. These constraints on regression coefficients reflect the prior information and structure, which can help us to find the optimal parameters with the given information. To incorporate the prior information in the modeling process, we add linear constraints to the penalized spatial logistic autoregressive model. As far as we are aware, no previous research has investigated penalized spatial logistics autoregressive models with linear constraints. Thus, in this paper, we will study a penalized spatial logistic autoregressive model with linear constraints. For the spatial logistic regression model (4), we estimate β by solving the following optimization

(1) $$\begin{array}{c} \min-\ln\left[L\left(\beta ,\rho \right)\right]+2n\sum_{j}^{p}p_{\lambda }\left(\left|\beta_{j}\right|\right)\\ \;\mathrm{subject}\;\mathrm{to}\;\;C\beta \leq d\;E\beta =f \end{array}$$

where L(β,ρ) is the likelihood function, ρ∈R, $\beta \in R^{p}$, *n* is the sample size, $p_{\lambda }\left(\cdot \right)$ is the penalty function, and $C\in R^{q\times p},d\in R^{q},E\in R^{s\times p}$, and $f\in R^{s}$ are determined concretely according to the experience and knowledge of practical problems. In this paper, our contribution is summarized as follows:

1. Propose a penalized spatial logistics autoregression with linear constraints. These constraints contain the prior information and structure, which can improve the robustness of the model and help us to find the optimal parameters. Thus, the model performs better in the variable selection and estimation under a high-dimensional data space.
2. Provide the formula for degrees of freedom, and then construct the model selection criteria to select the optimal tuning parameter.
3. Simulation results show that the performance of the proposed method is more explanatory and reasonable than penalized quasi-maximum likelihood without linear constraints, and an empirical application illustrates the usefulness of the methods in practical work. The effectiveness of the penalized quasi-maximum likelihood with linear constraints algorithm is demonstrated.

The following is how the paper is arranged. In Section 2, we introduce the general form of the problem we study and present our penalized quasi-maximum likelihood without linear constraints algorithm. The formula for degrees of freedom using Stein’s unbiased risk estimation (SURE) lemma is derived in detail in Section 3. Some Monte Carlos results on the performance of the proposed method are discussed in Section 4. Section 5 shows our method for analyzing real data sets. Section 6 presents the conclusions.

## 2. Models

### 2.1. Spatial Autoregressive Model (SAR)

Think of a network that has *n* nodes. The matrix $A\in R^{n\times n}$ can be used to characterize how the network is structured. Define $a_{ij}=1$ when node *i* follows node *j*, and $a_{ij}=0$ otherwise. With a n×1 vector of observations on the dependent variable *Y* and a n×p matrix of regressors *X*, we can establish the following SAR [3] model:

(2) Y=ρWY+Xβ+ϵ,

where ρ∈R is network autocorrelation coefficient and $\beta ={\left(\beta_{1},\ldots ,\beta_{p}\right)}^{T}\in R^{p}$ is the regression coefficient vector. *W* is the row-normalized version of *A* such that $w_{ij}=a_{ij}/\sum_{j=1}^{n}a_{ij}$. Let $\theta ={\left(\rho ,\beta^{T}\right)}^{T}\in R^{p+1}$ and denote $\epsilon ={\left(\epsilon_{1},\ldots ,\epsilon_{n}\right)}^{T}$ as the error vector of independent disturbances with mean zero and finite variances $\sigma^{2}$.

Denote G=I−ρW,S=Y−ρWY−Xβ. Then the SAR model’s log-likelihood function is shown as follows:

(3) $$\ln L\left(\theta ,\sigma^{2}\right)=-\frac{n}{2}\ln\left(2\pi \right)-\frac{n}{2}\ln\sigma^{2}+\ln\left|G\right|-\frac{1}{2\sigma^{2}}S^{T}S.$$

### 2.2. Spatial Logistic Regression Model

The spatial logistic regression model is a combination of the spatial autoregressive model and the logistic regression model. Binary classification or multi-classification are both acceptable response variables for a logistic regression model. However, we solely take into account the case of the binary of the response variable.

The model (2) can be written as:

(4) $$\begin{array}{cc} y^{*} & ={\left(I-\rho W\right)}^{-1}\left(X\beta +\varepsilon \right)\\ \; & ={\left(I-\rho W\right)}^{-1}X\beta +{\left(I-\rho W\right)}^{-1}\varepsilon \\ \; & =HX\beta +e,e∼MVN(0,\Omega ) \end{array}$$

where MVN denotes the multivariate normal distribution. For simplicity, we use $y^{*}$ instead of Y where $H={\left(I-\rho W\right)}^{-1}$ is an (n×n) matrix, and define $e={\left(I-\rho W\right)}^{-1}\varepsilon$ as an (n×1) vector. Latent variable $y^{*}$ has a binary category which is defined as variable *y*:

(5) $$y_{i}=\left\{\begin{array}{c} 1,\;\mathrm{for}\;y_{i}^{*}>0\\ 0,\;\mathrm{for}\;y^{*}\leq 0. \end{array}\right.$$

Therefore, the probability of $P\left(y_{i}=1\right)$ and $P\left(y_{i}=0\right)$ is:

(6) $$\begin{array}{cc} P\left(y_{i}=1|X_{i}\right) & =P\left(y_{i}^{*}>0\right)\\ \; & =P\left({\left[HX\beta \right]}_{i}+e>0\right)\\ \; & =P\left(-e\leq {\left[HX\beta \right]}_{i}\right)\\ \; & =\frac{1}{1+\exp\left(-{\left[HX\beta \right]}_{i}\right)} \end{array}$$

(7) $$\begin{array}{cc} P\left(y_{i}=0|X_{i}\right) & =P\left(y_{i}^{*}\leq 0\right)\\ \; & =P\left({\left[HX\beta \right]}_{i}+e\leq 0\right)\\ \; & =P\left(-e>{\left[HX\beta \right]}_{i}\right)\\ \; & =1-P\left(-e\leq {\left[HX\beta \right]}_{i}\right)\\ \; & =1-\frac{1}{1+\exp\left(-{\left[HX\beta \right]}_{i}\right)} \end{array}$$

When we assume the mean value of e is 0 and the variance is Ω, then we get

(8) $$P\left(y_{i}=1\right)=\frac{1}{1+\exp\left(-\frac{{\left[HX\beta \right]}_{i}}{\Omega_{ii}}\right)}$$

where $\Omega_{ii}$ is the diagonal element of Ω, which is formed as $\Omega ={\left[{\left(I-\rho W\right)}^{\prime }\left(I-\rho W\right)\right]}^{-1}$. The same idea applies to $P\left(y_{i}=0\right)$.

The parameter estimation of spatial logistic regression can be obtained by maximum likelihood estimation (MLE). The parameter is estimated by maximizing the likelihood function of random variable $y_{i}$, which follows a Bernoulli distribution:

(9) $$L\left(\beta ,\rho \right)=\prod_{i=1}^{n}\left[{\left(\frac{1}{1+\exp\left(-\frac{{\left[HX\beta \right]}_{i}}{\Omega_{ii}}\right)}\right]}^{y_{i}}{\left[1-\frac{1}{1+\exp\left(-\frac{{\left[HX\beta \right]}_{i}}{\Omega_{ii}}\right)}\right]}^{1-y_{i}}\right)$$

Then, the natural log(ln) is used to transform the likelihood function as follows:

(10) $$\begin{array}{cc} \ln[L(\beta ,\rho )] & =\sum_{i=1}^{n}y_{i}\ln[\left[1+\exp\left(-\frac{{\left[HX\beta \right]}_{i}}{\Omega_{ii}}\right)\right]+\sum_{i=1}^{n}\left(1-y_{i}\right)\ln\left[1-\frac{1}{[1+\exp\left(-\frac{{\left[HX\beta \right]}_{i}}{\Omega_{ii}}\right)}\right] \end{array}$$

To estimate β and ρ, we use the maximization formula (10), then define

$$\left(\hat{\beta },\hat{\rho }\right)=\arg\max_{\left(\beta ,\rho \right)}\ln\left[L\left(\beta ,\rho \right)\right].$$

## 3. Main Results

### 3.1. Variable Selection with Linear Constraints

In many application fields, prior knowledge can be formulated as constraints on parameters to improve the effectiveness of variable selection and estimation. In this section, we consider the variable selection of the spatial logistic regression model with linear constraints.

We will concentrate on the variable selection for the spatial logistic regression model with linear constraints, that is

(11) $$\min-\ln\left[L\left(\beta ,\rho \right)\right]+2n\sum_{j=1}^{p}p_{\lambda }\left(\left|\beta_{j}\right|\right)\;\;s.t\;C\beta \geq d,E\beta =f.$$

where ρ∈R, $\beta \in R^{p}$, *n* is the sample size, and $C\in R^{q\times p}$, $d\in R^{q}$, $E\in R^{s\times p}$, and $f\in R^{s}$ are determined concretely according to practical knowledge and experience. $p_{\lambda }\left(•\right)$ is the penalty function, and the shrinkage degree of the penalty is determined by the tuning parameter λ in the penalty term. There are many popular choices for the penalty function $p_{\lambda }\left(•\right)$ in the statistics literature: (1) the LASSO penalty with $p_{\lambda }\left(t\right)=\lambda \left|t\right|$; (2) the SCAD penalty with $p_{\lambda }\left(t\right)=$$\lambda \int_{0}^{\left|t\right|}\min\left\{1,{\left(a-t/\lambda \right)}_{+}/\left(a-1\right)\right\}dt,a>2$ where $v_{+}$ denotes its positive part, that is, vI(v≥0); (3) the MCP with $p_{\lambda }\left(t\right)=\lambda \int_{0}^{\left|t\right|}{\left(1-t/\left(\lambda a\right)\right)}_{+}dt,a>1$. Penalty functions can provide estimators with three properties which include unbiasedness, sparsity, and continuity according to Fan and Li (2001) [23].

LASSO is not unbiased, and MCP calculation is relatively complex. Fan and Li (2001) [23] demonstrated the oracle properties for the SCAD in the variable selection aspect, and pointed out that the LASSO penalty does not possess the oracle properties. Compared with ridge regression, the SCAD penalty method reduces the prediction variance of the model. Moreover, the SCAD penalty method outperforms the LASSO penalty ones, which reduces the deviation of parameter estimation. Thus, we choose to use the SCAD penalty here.

### 3.2. Selection of the Tuning Parameter

We decided to choose the SCAD [23] penalty, relying on the analyses mentioned above. The penalty function is defined as:

(12) $$p_{\lambda }\left(|\beta |\right)=\left\{\begin{array}{cc} \lambda \left|\beta_{j}\right|, & 0\leq \left|\beta_{j}\right|<\lambda ,\\ -\left({\left|\beta_{j}\right|}^{2}-2a\lambda \left|\beta_{j}\right|+\lambda^{2}\right)/\left(2a-2\right), & \lambda \leq \left|\beta_{j}\right|<a\lambda ,\\ \left(a+1\right)\lambda^{2}/2, & \left|\beta_{j}\right|\geq a\lambda , \end{array}\right.$$

where λ≥0 and a>2 are tuning parameters. Here, *a* is usually taken to be 3.7, a fact that is elucidated in the work of Fan and Li (2001) [23]. At the same time, they have also shown that λ determines the shrinkage strength of parameter estimation. In this paper, the selection of tuning parameter λ by Bayesian information criterion (BIC) is also related to degrees of freedom.

The number of degrees of freedom measures the number of effective parameters in the regression model and the complexity of the model. It plays an important role in model assessment and selection. There are different ways to measure degrees of freedom. Assume that *y* follows a distribution $y∼(\mu ,\sigma^{2})$, where μ is the true mean and $\sigma^{2}$ is the variance. Ye(1998) [25] and Efron(2004) [26] defined the number of degrees of freedom as

(13) $$\mathrm{df}\left(\hat{\mu }\right)=\frac{1}{\sigma^{2}}\sum_{i=1}^{n}\mathrm{cov}\left(y_{i},{\hat{\mu }}_{i}\right).$$

where $\hat{\mu }\left(y\right)=\hat{y}=X^{*}\hat{\beta }$ is the fitted response for $y\in R^{n}$.

Under the framework of Stein’s unbiased risk estimation (SURE) theory (Stein (1981) [27]), $cov\left(y_{i},{\hat{\mu }}_{i}\right)$ can be estimated by $\sigma^{2}E\left[\frac{\partial \hat{\mu_{i}}}{\partial y_{i}}\right]$, if $\hat{\mu }\left(y\right)$ is continuous and almost differentiable. Then the expression for degrees of freedom of fitted $\hat{\mu }$ can be calculated as

(14) $$\mathrm{df}\left(\hat{\mu }\right)=E\left[\sum_{i=1}^{n}\frac{\partial {\hat{\mu }}_{i}}{\partial y_{i}}\right].$$

to apply it. We need to assume that the response is normally distributed, that is, $y∼N\left(\mu ,\sigma^{2}I_{n}\right)$.

Degrees of freedom are used effectively while selecting the tuning parameter λ. In this study, the model selection criteria will be based on the Bayesian information criterion (BIC) (Schwarz (1978) [28]):

(15) $$\mathrm{BIC}\left(\lambda \right)=\ln\left(\frac{1}{n}\sum_{i=1}^{n}\delta_{\tau }\left(y_{i}-{\hat{t}}_{i,\lambda }\right)\right)+\frac{\ln n}{n}df\left({\hat{t}}_{\lambda }\right).$$

Since we have calculated the corresponding $\hat{\rho }\left(\lambda \right)$ and $\hat{\beta }\left(\lambda \right)$ at each tuning parameter λ, we can use the corresponding fitted value $\hat{t}\left(\lambda \right)$ and degrees of freedom $df(\hat{t}\left(\lambda \right))$ to select the optimal λ that minimizes BIC(λ).

## 4. Simulation Studies

### 4.1. Simulation Experiment Design

In the simulation experiment, we test the performance of the model through Monte Carlo simulation. The random sample is generated by model (2.1) combined with model (2.7), in which the covariate is considered when the (q+3)-dimensional normal distribution with zero mean and covariance matrix $\left(\sigma_{ij}\right)$, where $\sigma_{ij}=0.5^{\left|i-j\right|}$. Therefore, *X* is an n×(q+3) matrix. In the following simulation, we set the number of samples n∈{60,90,120}, and the number of insignificant covariates q∈{5,10,25}. In this paper, we show the cases of q=5 and q=10 in the simulation results.

In the spatial autoregressive model, the network autocorrelation coefficient ρ is generated by the uniform distribution on the interval $\left[\rho_{1}-1,\rho_{1}+1\right]$, where $\rho_{1}\in \left\{0.8,0.5,0.2\right\}$. Define the spatial weight matrix $W=I_{R}⊗B_{m}$, where $B_{m}=\left(1/\left(m-1\right)\right)\left(1_{m}\cdot 1_{m}^{T}-I_{m}\right)$, and ⊗ is the Kronecker product. Denote $1_{m}$ as the *m*-dimensional column vector of 1. In the simulation experiment, we consider m=3, and differet values of *R*, where R∈{20,30,40}. The regression coefficients are set to $\beta ={\left(\beta_{1},\beta_{2},\beta_{3},0_{q}\right)}^{T}$, where $\left(\beta_{1},\beta_{2},\beta_{3}\right)$ is generated from the 3-dimensional normal distribution with the mean vector of (3,2,1.6), and the covariance matrix is $0.001I_{3}$.

To simplify the calculation, the regression coefficients are set to ${\left(3,2,1.6,0_{q}\right)}^{T}$, where $0_{q}$ is a *q*-dimensional zero vector. The response variable is given by the following formula:

(16) $$y^{*}={\left(I_{n}-\rho W\right)}^{-1}\left(X\beta +\varepsilon_{n}\right)$$

Then use the following formula to convert the response variable into a category variable:

(17) $$Y_{i}=\left\{\begin{array}{c} 1,\;\mathrm{for}\;y_{i}^{*}>0\\ 0,\;\mathrm{for}\;y_{i}^{*}\leq 0. \end{array}\right.$$

Thus, we can obtain the binary classification of the response variable *Y*. For purpose of confirming the robustness of the model, the error terms $\varepsilon_{i}$s are independently generated from the following two distributions: (a) normal distribution $\varepsilon_{i}∼N\left(0,\sigma^{2}I_{n}\right)$, denoted as $\varepsilon_{0}$ and (b) Gaussian mixture distribution $\varepsilon_{i}∼0.5N\left(-1,2.5^{2}\right)+0.5N\left(1,0.5^{2}\right)$, denoted as $\varepsilon_{1}$. $\sigma^{2}$ is generated by the uniform distribution on the interval $\left[\sigma_{1}-0.1,\sigma_{1}+0.1\right]$, where $\sigma_{1}\in \left\{1,2\right\}$. In the simulation experiment, we set σ=1.5.

In order to verify that the effect of the model with linear constraints is better, we will compare it with the model without constraints. In one case, we can set the constraints as:

(18) $$\begin{array}{cc} \; & \beta_{3}+\beta_{6}=1.6\\ \; & \beta_{1}+\beta_{5}=3\\ \; & \beta_{1}+\beta_{3}\geq 4\\ \; & \beta_{2}+\beta_{6}\leq 2.5 \end{array}$$

Obviously, we can find *C*, *d*, *E*, and *f*.

### 4.2. Evaluation Indicators

According to the above simulation experiments, we set the number of Monte Carlo repetitions at 1000. We define the following three indicators to evaluate the performance of variable selection in different settings.

**Correct**: the average number of coefficients of the true zeros correctly set to zero;

**Incorrect**: the average number of coefficients of the true nonzeros incorrectly set to zero;

**ME**: the mean error between the true value and the estimate, which is defined by:

(19) $$\frac{1}{1000}\sum_{i=1}^{1000}{∥\theta_{i}-{\hat{\theta }}_{i}∥}_{1}$$

### 4.3. Simulation Results

Table 1 and Table 2 show the results of models with linear constraints without linear constraints, respectively. The constrained model is recorded as "Const”, and the unconstrained model is recorded as “Unconst”. The results in Table 1 clearly show that the spatial logistic model performs better with linear constraints, which also confirms the effectiveness of our model. Most significantly, when $\rho_{1}=0.8$, the error of the models with constraints and without constraints are very different, which shows that when the spatial effect is strong the constraints can greatly improve the accuracy of model parameter estimations. Moreover, we find that the effect of the model tends to become bad by increasing the network autocorrelation coefficient $\rho_{1}$, which indicates the importance of the spatial effect. Moreover, by setting two types of errors, we observe that the model has good robustness. Additionally, in most cases, the model with an error term of $\varepsilon_{1}$ performs better than the one with an error term of $\varepsilon_{0}$. Similarly, with the increase in sample size n, the incorrect rate of variable selection and the estimation error both decrease. This situation is in line with our prediction of the effect of the model. Moreover, it can be seen in Table 1 that the mean error is minimum when *n* = 120, $\rho_{1}$ = 0.2, and $\varepsilon =\varepsilon_{1}$, which also confirms our analysis above.

In Table 2, by increasing the dimension to *q* = 10, we find that with the increase in the network autocorrelation coefficient $\rho_{1}$, the effect of the model becomes bad. Compared to the case of *q* = 5, we find an increase in the mean error of estimation. After analysis, the possible reason is that the proportion of data size to dimension is bad, that is, the dimension of the sample is higher than the sample size. suppose the network complexity increases, it will have a certain negative impact on the effect of variable selection. For high-dimensional samples, the model results can be optimized by increasing the sample size. Simultaneously, according to the simulation results, compared with the unconstrained case, the spatial logistic model has stronger robustness, higher accuracy, and lower estimation error rate.

We compare the variable selection using the SCAD penalty and LASSO penalty under constraints. The simulation results are shown in Table 3. We can clearly observe that in the case where $\rho_{1}=0.2$ and $\rho_{1}=0.5$, the performance of the SCAD penalty is significantly better than the LASSO penalty, which is shown in higher correct selection rate and lower estimation error. As the network autocorrelation coefficient increases to $\rho_{1}=0.8$, the correct rate of variable selection of the SCAD penalty is higher than the LASSO penalty, while the incorrect rate of the LASSO penalty is lower than the SCAD penalty. Additionally, the estimation error between them is not much different. The reason may be that the tuning parameter λ in the LASSO penalty is too large and the shrinkage strength is stronger.

## 5. Real Data Example

In this section, we provide a real-world example to demonstrate the performance of the variable selection procedure proposed in this paper for spatial logistic regression models with linear constraints.

### 5.1. The Land Area Utilization Data

Land area utilization is analyzed by the spatial logistic model. The data set is different types of land area data from 48 states in the United States from 1954 to 2012 (recorded every five years). The dependent variables are binary, with “1” denoting a low land utilization rate, which means that most of the land has not been properly developed, and “0”, denoting a high land utilization rate, which means that most of the land has been efficiently developed and exploited. As for the independent variables, there are eight properties, which are Cropland used for crops, Cropland used for pasture, Cropland idled, Grassland pasture and range, Forest-use land grazed, Land in rural transportation facilities, Land in urban areas, and Other idle land (shown in Table 4).

### 5.2. Variable Selection and Estimation

For the above land area utilization data sets, we constructed a spatial logistic autoregressive model. We use the land utilization rate as a response variable, and take eight variables, Cropland used for crops, Cropland used for pasture, Cropland idled, Grassland pasture and range, Forest-use land grazed, Land in rural transportation facilities, Land in urban areas, and Other idle land, as the independent variables.

According to theoretical knowledge, the idle area of cropland and other idle land areas have a significant impact on the probability of classification results. Through the fitting of the model, the following two cases are considered: the first is the parameter estimation without constraints, and the other is the parameter estimation with linear constraints, as shown in Table 5 and Table 6.

According to the results of parameter estimation based on the spatial logistic model, it is found that the performance of variable selection is not obvious under unconstrained conditions. Among them, “Forest-use land grazed” and “Land in rural transportation facilities” have little impact on land use efficiency, which can be almost ignored. However, “Other idle land” has a great influence on the classification effect. Considering the greater relationship between land use area and idle land area, the performance of model selection is greatly improved with constraints. According to the table below, it is found that “Cropland idle” and “Other idle land” have a great impact on the classification results, and the fitting parameters of other attributes are very small and can be ignored.

## 6. Conclusions

In the paper, we obtain a spatial logistic model from the spatial autoregressive model (SAR) and logistic regression model. In order to improve the accuracy of the model, we take the prior information into account, and finally, propose a variable selection method with linear constraints for the spatial logistic model. According to the simulation results, by comparing the constrained and unconstrained variable selection models, we find that the performance of variable selection is more stable with the increase in sample size in the case of limited samples. When we fix the sample size at a constant value, the performance of the model tends to improve with the increase in network complexity. At the same time, the model has strong robustness to noise. In order to verify the superiority of the SCAD penalty, we compare the performance of the SCAD and LASSO penalties in the case of the linear constraint model and find that the SCAD penalty has a better effect on variable selection.

In practical application, most data show the characteristics of small sample size and high dimensions. For purpose of verifying the wide adaptability of the model, we verify that the proposed model can be effectively applied to the data set of high-dimensional and small samples through simulation experiments. However, we find that in this case, when the network complexity is very high, the performance effect of the model is not very good. The sample size being too small might be the cause of this.

## Figures and Tables

**Table 1:** Simulation results of the SCAD penalty model with constraints and without constraints (q=5).

Method		n = 60, q = 5		n = 90, q = 5		n = 120, q = 5	
		Const	Unconst	Const	Unconst	Const	Unconst
$\rho_{1}=0.2$	Correct	4.703	4.637	4.755	4.809	4.789	4.812
$\varepsilon_{0}$	Incorrect	0	0.189	0	0.099	0	0.068
	ME	0.323	4.052	0.290	3.013	0.273	2.542
$\rho_{1}=0.2$	Correct	4.780	4.634	4.783	4.782	4.784	4.837
$\varepsilon_{1}$	Incorrect	0	0.114	0	0.050	0	0.032
	ME	0.314	5.651	0.288	4.789	0.262	3.959
$\rho_{1}=0.5$	Correct	4.682	4.421	4.752	4.589	4.701	4.664
$\varepsilon_{0}$	Incorrect	0	0.135	0	0.062	0	0.044
	ME	0.402	6.965	0.331	5.434	0.319	4.995
$\rho_{1}=0.5$	Correct	4.692	4.385	4.675	4.548	4.700	4.672
$\varepsilon_{1}$	Incorrect	0	0.084	0	0.050	0	0.029
	ME	0.374	9.335	0.338	7.735	0.319	7.073
$\rho_{1}=0.8$	Correct	3.894	2.998	4.101	3.619	4.179	3.809
$\varepsilon_{0}$	Incorrect	0.075	0.065	0.021	0.018	0.018	0.015
	ME	2.362	45.941	1.442	29.596	1.093	23.867
$\rho_{1}=0.8$	Correct	3.865	2.837	4.126	3.542	4.174	3.876
$\varepsilon_{1}$	Incorrect	0.074	0.052	0.016	0.014	0.028	0.007
	ME	2.243	51.830	1.365	35.213	1.092	29.309

**Table 2:** Simulation results of the SCAD penalty model with constraints and without constraints (q=10).

Method		n = 60, q = 10		n = 90, q = 10		n = 120, q = 10	
		Const	Unconst	Const	Unconst	Const	Unconst
$\rho_{1}=0.2$	Correct	9.255	8.740	9.374	9.363	9.571	9.614
$\varepsilon_{0}$	Incorrect	0	0.166	0	0.074	0	0.036
	ME	0.383	3.713	0.317	3.072	0.281	2.487
$\rho_{1}=0.2$	Correct	9.777	9.282	9.861	9.178	9.642	9.546
$\varepsilon_{1}$	Incorrect	0	0.102	0	0.021	0	0.015
	ME	0.328	5.679	0.293	4.697	0.262	4.134
$\rho_{1}=0.5$	Correct	8.036	7.220	8.344	7.970	8.598	8.298
$\varepsilon_{0}$	Incorrect	0	0.133	0	0.052	0	0.022
	ME	0.671	6.641	0.493	5.272	0.446	4.853
$\rho_{1}=0.5$	Correct	9.571	6.632	8.395	8.160	8.690	8.576
$\varepsilon_{1}$	Incorrect	0	0.117	0	0.034	0	0.009
	ME	0.414	9.101	0.488	7.217	0.419	6.829
$\rho_{1}=0.8$	Correct	5.688	4.049	5.889	5.204	6.221	9.755
$\varepsilon_{0}$	Incorrect	0.129	0.070	0.071	0.020	0.080	2.937
	ME	10.522	57.696	6.465	34.675	5.039	6.697
$\rho_{1}=0.8$	Correct	5.669	4.046	6.019	5.152	6.194	9.759
$\varepsilon_{1}$	Incorrect	0.134	0.043	0.076	0.023	0.079	2.935
	ME	9.901	57.584	6.345	37.509	4.627	6.690

**Table 3:** Simulation results of the constrained model with the SCAD penalty and LASSO penalty.

Method		n = 60, q = 5		n = 90, q = 5		n =120, q = 5	
		SCAD	LASSO	SCAD	LASSO	SCAD	LASSO
$\rho_{1}=0.2$	Correct	4.703	3.496	4.755	3.495	4.789	3.483
$\varepsilon_{0}$	Incorrect	0	0	0	0	0	0
	ME	0.323	2.182	0.290	2.162	0.273	2.152
$\rho_{1}=0.2$	Correct	4.780	3.482	4.783	3.472	4.784	3.397
$\varepsilon_{1}$	Incorrect	0	0	0	0	0	0
	ME	0.314	2.157	0.288	2.146	0.262	2.096
$\rho_{1}=0.5$	Correct	4.682	3.633	4.752	3.646	4.701	3.625
$\varepsilon_{0}$	Incorrect	0	0	0	0	0	0
	ME	0.402	2.227	0.331	2.212	0.319	2.219
$\rho_{1}=0.5$	Correct	4.692	3.605	4.675	3.683	4.700	3.624
$\varepsilon_{1}$	Incorrect	0	0	0	0	0	0
	ME	0.374	2.210	0.338	2.222	0.319	2.221
$\rho_{1}=0.8$	Correct	3.894	3.516	4.101	3.516	4.179	3.509
$\varepsilon_{0}$	Incorrect	0.075	0	0.021	0	0.018	0
	ME	2.362	2.327	1.442	2.285	1.093	2.307
$\rho_{1}=0.8$	Correct	3.865	3.478	4.126	3.538	4.174	3.485
$\varepsilon_{1}$	Incorrect	0.074	0	0.016	0	0.028	0
	ME	2.243	2.348	1.365	2.274	1.092	2.272

**Table 4:** Summary of predictor variables for the land area utilization model.

Variable Name	Description
CLand_C	Cropland used for crops
CLand_P	Cropland used for pasture
CLand_I	Cropland idled
Grass_P	Grassland pasture and range
Land_G	Forest-use land grazed
Land_T	Land in rural transportation facilities
Land_U	Land in urban areas
Land_I	Other idle land

**Table 5:** Parameter estimation results without constraints.

Year	CLand_C	CLand_P	CLand_I	Grass_P	Land_G	Land_T	Land_U	Land_I
1954	0	0	0.982	0.423	0	0	0	0.602
1959	0	1.700	0	−1.740	0	0	0.3050	1.27
1964	0	1.28	0	−0.615	0	0	0	0.892
1969	0	1.55	0.715	−1.49	0	−0.385	0	1.06
1974	0.854	1.52	1.39	−2.26	0	−0.596	−0.651	1.52
1978	0.663	0	1.90	−1.72	−0.249	−0.287	0.614	1.26
1982	0.302	1.45	1.12	−1.12	0	0	−0.984	1.14
1987	0	1.15	0.664	0	0	0	−1.11	1.25
1992	0.981	0	0.174	2.82	0	−0.949	−2.41	1.80
1997	0.568	0	0.262	2.75	0	−0.877	−1.93	1.87
2002	2.42	0	1.61	−1.21	0	−1.00	0	2.39
2007	1.30	0.852	1.76	−1.20	0	0	−0.383	2.47
2012	0	0	2.58	−0.468	0	−0.799	0	1.02

**Table 6:** Parameter estimation results with linear constraints.

Year	CLand_C	CLand_P	CLand_I	Grass_P	Land_G	Land_T	Land_U	Land_I
1954	0	0	0.982	0.419	0	0	0	0.604
1959	0	0	0.660	−0.298	0.600	−0.464	0	1.16
1964	0	0	0	0.663	0	0	0	0.691
1969	0	0	1.03	−0.3.16	0.311	−0.452	0	0.814
1974	0	0	1.74	−1.21	0.640	−0.639	0	1.10
1978	0	0	1.83	−0.741	0	0	0	1.41
1982	0	0	0.922	0	0.604	−0.499	0	0.884
1987	0	0	0.903	0	0.585	−0.547	0	0.945
1992	0	0	0.514	0.596	0.447	−0.545	0	0.981
1997	0	0	0.586	0.770	0.408	−0.610	0	1.05
2002	0	0	1.48	0	0	0	0	2.24
2007	0	0	2.05	0	0	0	0	1.04
2012	0	0	1.76	0	0	0	0	0.694

## Data Availability

Not applicable.
